# Testing ATRA and MEK inhibitor PD0325901 effectiveness in a nude mouse model for human MPNST xenografts

**DOI:** 10.1186/s13104-018-3630-0

**Published:** 2018-07-28

**Authors:** Susan Fischer-Huchzermeyer, Levan Chikobava, Verena Stahn, Monique Zangarini, Philip Berry, Gareth J. Veal, Volker Senner, Victor F. Mautner, Anja Harder

**Affiliations:** 1 Institute of Neuropathology, University Hospital Münster, Münster, Germany; 2 Northern Institute for Cancer Research, Newcastle University, Newcastle upon Tyne, UK; 3 Clinics and Polyclinics of Neurology, University Hospital Hamburg-Eppendorf, Hamburg, Germany; 4 Institute of Pathology, Health Care Center, Brandenburg Hospital, Brandenburg Medical School Theodor Fontane, Brandenburg, Germany

**Keywords:** Malignant peripheral nerve sheath tumors (MPNST), Neurofibromatosis type 1 (NF1), Nude mouse model, All-*trans* retinoic acid (ATRA), MEK inhibitor (MEKi), S462, T265, PD0325901, Xenograft model

## Abstract

**Objective:**

Malignant peripheral nerve sheath tumors (MPNST) are aggressive sarcomas characterized by high recurrence rates and early metastases. These tumors arise more frequently within neurofibromatosis type 1 (NF1) and present with resistance during standard chemotherapy leading to increased mortality and morbidity in those patients. In vitro all-*trans* retinoic acid (ATRA) and MEK inhibitors (MEKi) were shown to inhibit tumor proliferation, especially when applied in combination. Therefore, we established a nude mouse model to investigate if treatment of xenografts derived from NF1 associated S462 and T265 MPNST cells respond to ATRA and the MEKi PD0325901.

**Results:**

We demonstrated that human NF1 associated MPNST derived from S462 but not T265 cells form solid subcutaneous tumors in Foxn1 nude mice but not in Balb/c, SHO or Shorn mice. We verified a characteristic staining pattern of human MPNST xenografts by immunohistochemistry. Therapeutic effects of ATRA and/or MEKi PD0325901 on growth of S462 MPNST xenografts in Foxn1 nude mice were not demonstrated in vitro, as we did not observe significant suppression of MPNST growth compared with placebo treatment.

**Electronic supplementary material:**

The online version of this article (10.1186/s13104-018-3630-0) contains supplementary material, which is available to authorized users.

## Introduction

Neurofibromatosis type 1 (NF1) is a risk factor for the development of malignant peripheral nerve sheath tumors (MPNST), which are associated with poor prognosis due to high recurrence and early metastases [1, 2]. In NF1, MPNST tend to arise from plexiform neurofibromas as a consequence of *NF1* mutations and LOH in Schwann cells, leading to activation of Ras signaling [3]. Treatment involves surgical removal and chemotherapy. However, MPNST display significant resistance to standard chemotherapy [4, 5], therefore adapted therapies are necessary.

Aberrant MAPK cascade activation (Raf/Mek/Erk) resulting from neurofibromin inactivation is involved in MPNST formation. A recent study detected several MEK inhibitors (MEKi) to be active in NF1-associated MPNST [6] and the small molecule MEKi and multi kinase inhibitor sorafenib has shown anti-tumor properties in MPNST in vitro [6–8]. The effect of MEKi on MPNST can be increased by co-treatment with agents such as ATRA, BMP2, mTOR kinase inhibitors (AZD8055, RAD001), PAK1/2/3 inhibitors and photothermal therapy in vitro, as well as with PAK inhibitors and photothermal therapy in xenograft models [9–14]. Pre-clinical transgenic mouse models demonstrated efficacy of MEKi when admitted alone, however increased MEKi properties were seen when combined with RAD001 [8, 14, 15]. Unfortunately, studies testing sorafenib showed only minimal response in MPNST patients [16].

We recently identified a crucial role of retinoic acid in MPNST, suggesting a potential therapeutic option as shown in other cancers [9, 17, 18]. Combination therapy of ATRA is used not only to overcome resistance but also to enhance therapeutic effects. Thus, a combination of retinoic acid with interferon alpha 2a in progressive metastatic renal cell carcinoma, or with histone deacetylase-inhibitor valproic acid in refractory and high-risk acute myeloid leukemia, demonstrated beneficial effects [19, 20]. In neuroblastoma cells, inhibition of MAPK cascade downstream Ras has been shown to restore ATRA responsiveness [21, 22].

In the current study we have attempted to verify whether ATRA and MEKi, both alone and in combination, exhibit efficacy in a xenograft nude mouse model for human MPNST, as we have recently demonstrated in vitro [9, 17, 18]. A combination of ATRA and MEKi may provide a novel promising therapeutic approach for MPNST.

## Main text

### Materials and methods

#### Cell culture and colony formation assay

Human MPNST cell lines S462 and T265 were described previously [23–26]. Cells were cultured in DMEM (4.5 g/L glucose, 2 mM l-glutamine, 10% (v/v) FBS, 100 U/mL penicillin/streptomycin and 1 mM sodium pyruvate). Clonogenic assays were performed as described elsewhere with specifications: 300 cells per well were seeded in a 6-well plate and incubated for 14 days. [27] Following incubation, cells were washed with PBS and incubated with staining/fixation solution (6% glutaraldehyde/0.5% crystal violet/PBS) for 30 min. Colonies were defined as accumulation of > 50 cells. Images were taken per well (Olympus SZX12 microscope) using Adobe Photoshop CS5 (Adobe Systems Software Ireland Limited 2010), and colonies were counted using the cell counter tool of ImageJ (NIH United States 2014).

#### Xenotransplantation and ATRA quantification

Experiments were approved by the federal state authority of nature, environment and consumer protection of Nordrhein-Westfalen (“Landesamt für Natur, Umwelt und Verbraucherschutz Nordrhein-Westfalen“, LANUV) (27.08.2013, reference 84-02.04.2013.A275). Balb/c Nude (Balb/cAnNRj-Foxn1nu/Foxn1nu, Janvier), Foxn1 (Nu/Nu) Nude (Crl:NU-Foxn1nu, Charles River), SHO^®^ (SCID Hairless Outbred, Crl:SHO-PrkdcscidHrhr, Charles River) and Shorn (ShrN NOD SCID, NOD.Cg-PrkdcscidHrhr/NCrHsd, Harlan) mice were scheduled for xenotransplantations. Female mice were housed in single cages and all strains lacked hair and T cells. SHO^®^ and Shorn mice also lacked B cells, and Shorn mice additional partially lacked NK cells. We followed published criteria for generating xenografts.

At study initiation nude mice were 5–7 weeks old. A total of 5 × 10^6^ cells (T265, S462) within 30% Matrigel™ (Corning, GFR)/DMEM were implanted by subcutaneous injection (150 μL) into the right and/or left flank.

Blood was collected on day 21 of treatment, 2–3 h after oral application. Plasma was extracted immediately from blood by centrifugation (1000×*g*, 5 min). ATRA plasma levels were analyzed as described elsewhere [28].

#### Pharmacological treatment, tumor volume quantification and preparation

Daily oral treatment started 42 days after subcutaneous implantation over a period of 28 days. Ten mice were treated with ATRA (15 mg/kg) or PD0325901 (10 mg/kg). Intake was scored (1 = full intake, 0.5 = incomplete intake, 0 = no intake) and mean values were calculated. Tumor volume was measured three times a week using a digital caliper and calculated according to the formula: v = L × W^2^ (π/6) (L-longest diameter, W-width) [29].

Intra-cardiac perfusion was used for euthanization of mice. Under deep anaesthesia (1.8% (v/v) ketamine and 0.3% xylazine in PBS), peritoneal cavity, thorax, left heart ventricle and the right heart atrium were opened, and 10 mL PBS were injected into the ventricle. For fixation of mice tissues injection of 10 mL 4% paraformaldehyde followed PBS intra-cardiac perfusion. Tumors were dissected and stored in 4% PFA for 24 h followed by paraffin embedding. Tumors were dehydrated in increasing alcohol concentrations in a Tissue-Tek VIP system (Sakura) and embedded in paraffin. Immunohistochemistry of MPNST xenografts was performed using antibodies against S100 (monoclonal, mouse, 10 μg/mL, Ventana S100-4C4.9) to demonstrate human Schwann cell origin, anti-Ki67 (monoclonal, mouse, 0.4 μg/mL, Ventana Ki-67-30.9) to evaluate proliferation fraction, anti-CD34 (monoclonal, mouse, 0.8 μg/mL, Ventana QBEnd/10) to demonstrate vessel and stem cell formation, anti-p53 (monoclonal, mouse, Ventana Bp-53-11), anti-p16 (monoclonal, mouse, 1 μg/mL, Ventana 725-4713), anti-vimentin (vimentin, monoclonal, mouse, 2.5 μg/mL, Ventana V9 and anti-actin (monoclonal, mouse, 0.02 μg/mL, Cell Margue 1A4). Staining was visualized using the ultraView™ Universal Alkaline Phosphatase Red Detection Kit (Ventana, A-PAP method). Staining was performed in the automated staining system Ventana BenchMark XT according to the standard protocols.

#### Statistical analysis

ANOVA followed by post hoc *t* test was used to compare differences between groups. Kruskal–Wallis test was applied to compare groups of 4 mice for weight gain. A p value < 0.05 was defined to be significant. The SPSS program (IBM SPSS Statistics Standard) was applied.

### Results

We investigated four strains and cell lines T265 and S462. To determine tumor formation capability colony formation assays were performed and yielded 42 ± 6 colonies of T265 cells and 21 ± 5 colonies of S462 cells from 300 cells plated.

Once clonogenic potential had been demonstrated, we tested strains for tumor formation capability. 2 × 10^6^, 5 × 10^6^, and 10 × 10^6^ MPNST cells were subcutaneously transplanted into the right flank of Balb/c mice (6 groups of 4–5 mice). After 8 weeks tumor volumes did not reach 70 mm^3^. Differences with respect to cell number were not seen. To test other strains for xenotransplantation, 5 × 10^6^ MPNST cells were transplanted into the right and left flank (2 mice/strain, 2 transplants/mouse) of Foxn1, SHO and Shorn mice, and growth was monitored for 8 weeks. Transplants of T265 cells did not generate tumors in Foxn1 mice whereas SHO and Shorn mice developed one flank tumor each (2 tumors/4 transplants) with only 1 tumor gaining a volume > 70 mm^3^. Transplantation of S462 cells resulted in complete tumor development in all Foxn1 mice (4/4), and all tumors reached volumes > 70 mm^3^ 48 days after transplantation. SHO mice developed 3/4 tumors, but volumes did not reach 70 mm^3^. Only one tumor in Shorn mice (1/4) below 70 mm^3^ was seen. MPNST pattern of xenografts was confirmed histologically.

Beforehand, we had checked in Balb/c mice if application of ATRA and MEKi was tolerated and if oral application was sufficiently fast and complete. Oral administration of ATRA was prescribed by the veterinarian. Oral intake of MEKi is published in literature [15]. In our analysis intake of 0.2 g chocolate with ATRA was identical to chocolate with DMSO (placebo group) (mean intake: 1). Due to its strong flavor, the MEKi had to be served with 0.2 g peanut butter, still 1 mouse showed low intake (< 0.5). ATRA and MEKi treatment as well as combination was also-pre-checked to determine potential side effects in groups of 4 mice per agent as well as placebo (DMSO). We did not observe major side effects, in fact temporary unilateral eye irritation was the only impairment seen independently of treatment in all groups (sparing SHO mice) which did not meet the criteria for exclusion. None of the mice presented with significant weight loss (> 20%): by Kruskal–Wallis test we did not detect significant differences due to treatment regimens (placebo/chocolate, ATRA/chocolate, MEKi/peanut butter, control/normal diet). Macroscopy and histological examination of brain, spleen, kidney, stomach, liver and heart of all animals was without any pathology at the end of experiments in all animals.

From the above experiments, we selected S462 cells for transplantation into the right flank of Foxn1 mice. At day 42 after transplantation placebo, ATRA and MEKi, both alone and in combination, were administered until day 68. At day 42 mean tumor volume of the placebo group (n = 10) was 87.6 (± 31.4 SD) mm^3^, of the ATRA group (n = 9) was 106.2 (± 11.9 SD) mm^3^, and the MEKi group (n = 10) was 99.2 (± 51.3 SD).

ATRA plasma levels following ATRA administration were determined on day 21. Levels observed in the ATRA group were 2.76 µM (mean ± 2.07; range 0.41–5.84), compared to levels below the detection limit of 0.07 μM in the placebo group. These exposures in treated animals were consistent with levels of 1–5 μM observed in human acute promyelocytic leukemia patients.

Unexpectedly, Foxn1 mice of ATRA-MEKi combination group did not show sufficient food intake as we had trialed in Balb/c: Compared to placebo group (intake: 1 during the whole treatment period) ATRA/MEKi group showed low intake rates. We therefore exclude this group from experiments.

**Fig. 1:**
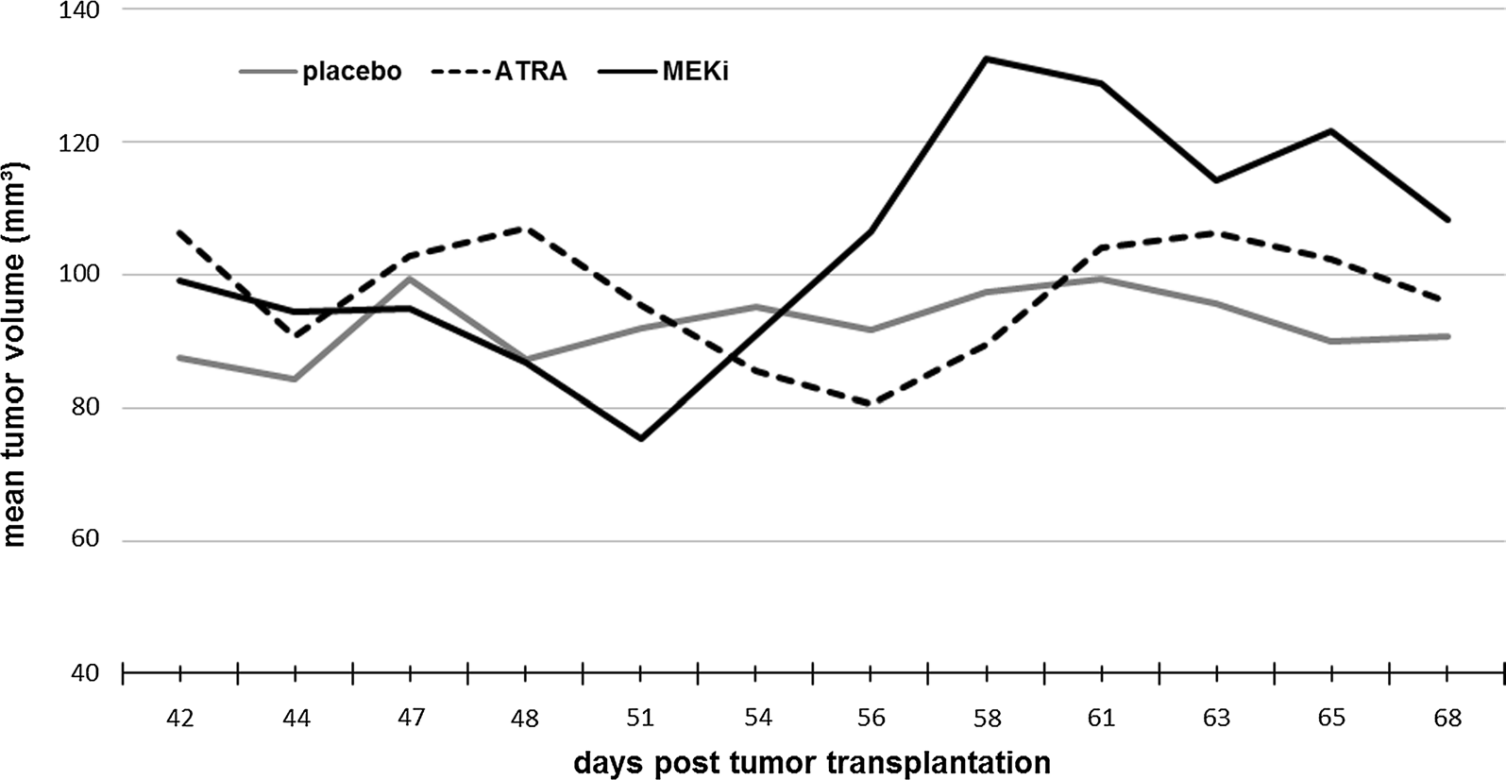
Growth characteristics (mean tumor volumes) of S462 MPNST xenografts are shown for ATRA and MEKi treatment and placebo

**Fig. 2:**
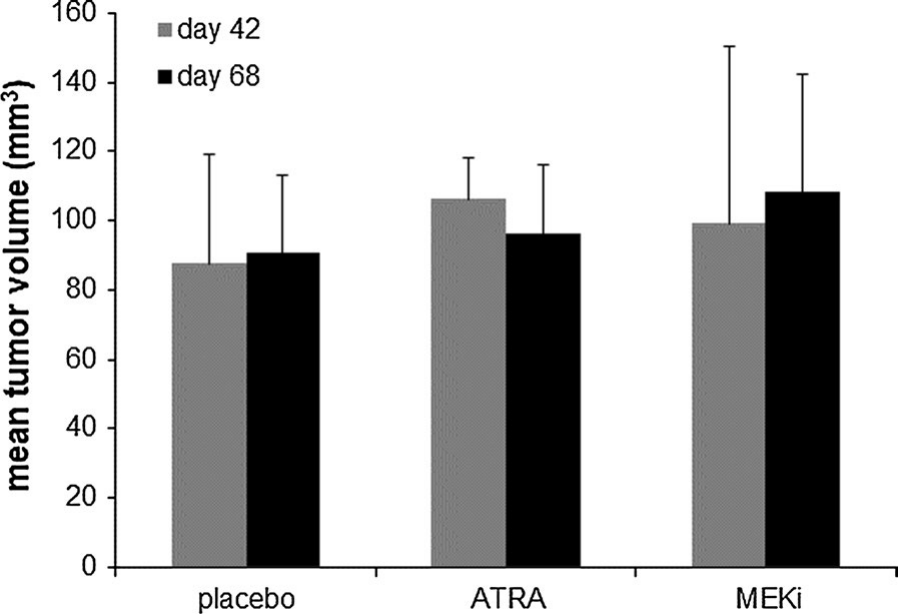
Growth characteristics of S462 MPNST xenografts in nude mice demonstrating tumor volumes at start (day 42) and end of therapy (day 68)

Mean tumor volume at the end of experiments (day 68) were as follows: 90.7 (± 22.2 SD) mm^3^ in the placebo group (n = 10), 96.0 (± 19.9 SD) mm^3^ in the ATRA group (n = 9), and 108.3 (± 34.1 SD) in the MEKi group (n = 10). All data are shown in Additional file 1. Comparing mean tumor volumes at the end of experiments revealed no statistically significant differences as compared to the untreated placebo group (Figs. 1, 2). Mean tumor volumes were higher in the ATRA group at the start of the experiments and a decrease in tumor volumes was seen following treatment, nevertheless differences between first and last day of treatment were statistically not significant. The animals treated with MEKi also did not show significant changes of tumor volumes. Principally, we defined tumor regression (that may have been observed independently from the poor tumor growth characteristics of the placebo) as ≥ 50% reduction of volume compared to volume at the start of treatment. However, no treatment group or single animals met this criterion.

**Fig. 3:**
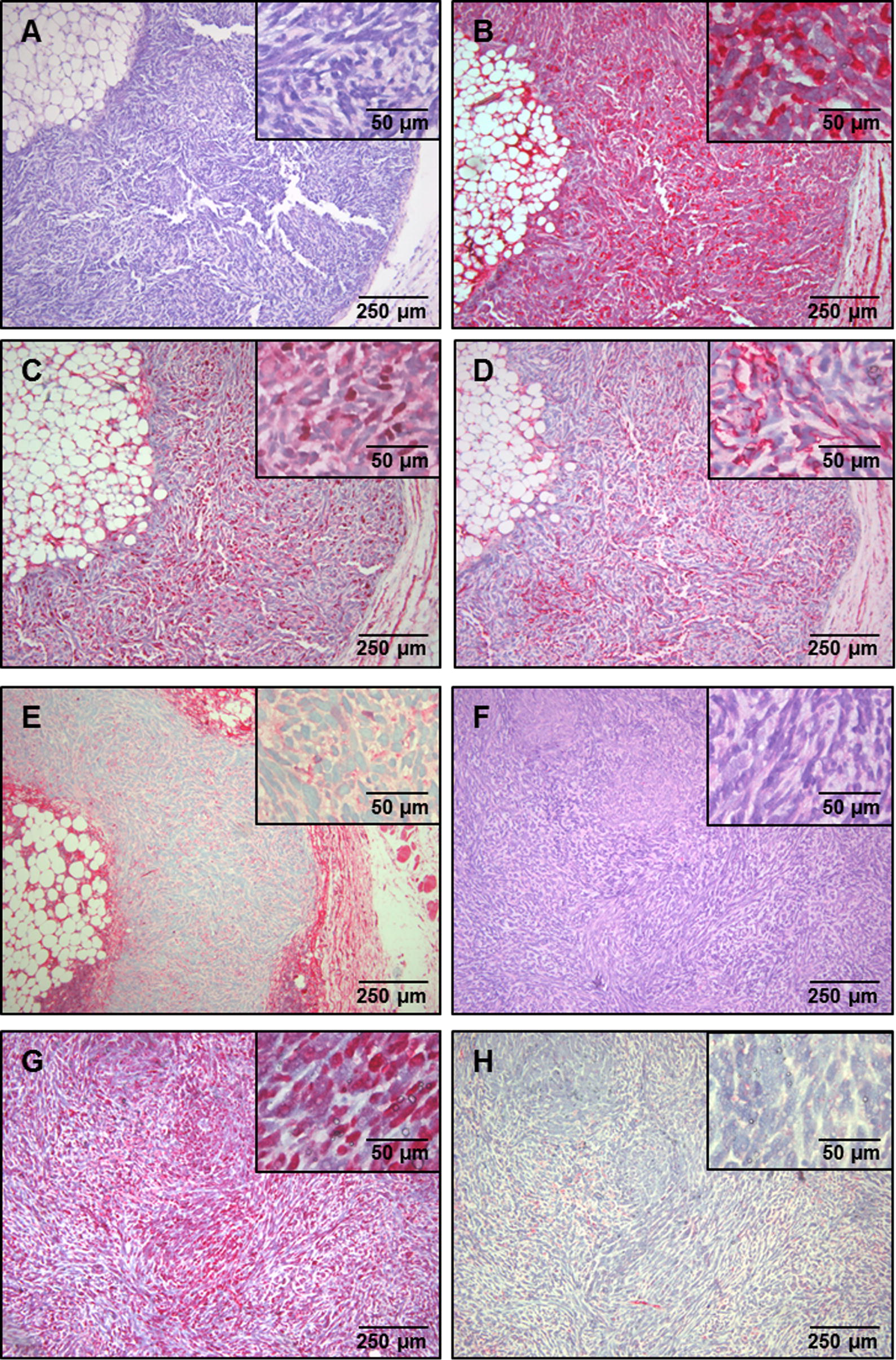
Histomorphological features S462 MPNST xenografts. **A** HE staining of a highly cellular MPNST xenograft. **B** Tumor cells stain positive S100 (nuclear and cytoplasmatic. **C** A high Ki-67 labelling index of 45% was determined by MIB-1 staining. **D** Staining for CD34 was found to be positive for single cells of the xenograft. **E** Staining for actin was present only in single cells. **F** HE staining of a hyper-cellular MPNST xenograft. **G** Strong nuclear p53 expression and **H** absence of p16 expression

Finally, S462 xenografts were subjected to HE stain and immunohistochemistry. Typical MPNST morphology was confirmed by neuropathological examination (Fig. 3). Marked inflammation was not seen. Areas of low cellular density and myxoid change or adipose tissue within tumors that were otherwise highly cellular indicating focal differentiation within the MPNST (Triton tumor-like) was identified in some cases. Expression of S100 and a strong nuclear expression of p53 were detected indicating additional *TP53* mutations in MPNST and no genotype change of the transplants. Nuclear expression of Ki-67 reached 40–45%. As MPNST typically acquire *CDKN2A* mutations transplanted S462 cells demonstrated loss of p16 as well. To exclude minor histopathological changes all tumors of animals were investigated using H&E staining and immunohistochemistry in this study, tumors of the main experiments were also cutted in 3 µm series for evaluation.

## Conclusions

In contrast to other in vivo studies, we observed that neither ATRA nor MEKi therapy significantly reduced growth of S462 derived xenografts in Fox1 nude mice. Due to several limitations we expect that tumor formation capability, agent administration, mouse strain and treatment period are critical parameters, that need to be exhaustedly examined before excluding MEKi and/or ATRA treatment as sufficient agents against MPNST.

## Limitations

- Reduced acceptance of oral administration led to exclusion of an important subgroup (ATRA/MEKi) from our study. Thus, intraperitoneal application that allows exact dose control may be more suitable in future experiments.
- Although tumor formation capability of S462 cells was reliably pre-tested in Foxn1 mice, tumor volumes did not reach values described in literature (750–2500 mm^3^) [15, 30, 31] in almost three studies with S462 cells or with other MPNST cell lines [29, 30, 32, 33]. Thus, selecting more suitable mouse strains and longer treatment periods may improve validity.
- Tumor growth was not seen in Balb/c mice at all, although those have been successfully used in several ATRA treatment studies for human tumor xenografts including medulloblastoma, melanoma and others [34, 35]. T265 and S462 MPNST cells seem not to bear sufficient tumor formation capacity in this otherwise well investigated strain. Thus, selection of other MPNST cell lines seems necessary to test the hypothesis (at least in Balb/c mice).
- Measuring small tumors with a caliper hampers significance. MRI or ultrasound may be more suitable to control tumor size.
- Insufficient growth characteristics may be due to Triton tumor-like differentiation within MPNST xenografts, however we cannot provide detailed investigations.

## Additional file

**Additional file 1.** Mean tumor volumes. Description of data: Mean volumes of transplanted MPNST (mm³) of two treatment groups (mice treated with ATRA and with MEKi) and of placebo group. Tumors were measured from day 7 until day 68 post transplantation. Treatment started at day 42. Average volumes and standard deviation over the whole group per day as well as difference of volumes between day 42 and 68 are given.

## Abbreviations

ATRA: all-*trans* retinoic acid
BMP2: bone morphogenetic Protein 2
DMEM: Dulbecco’s modified eagle’s medium
ERK: extracellular-signal regulated kinase
LOH: loss of heterozygosity
MAPK: mitogen-activated protein kinase
MEK: mitogen-activated protein kinase
MEKi: MEK inhibitor
mTOR: mechanistic target of rapamycin
MPNST: malignant peripheral nerve sheath tumor
NF1: neurofibromatosis type 1
PAK: p21-Activated Kinase
RAF: rapidly accelerated fibrosarcoma
RAS: rat sarcoma

## References

[1] Ducatman BS, Scheithauer BW, Piepgras DG, Reiman HM, Ilstrup DM (1986). Malignant peripheral nerve sheath tumors. A clinicopathologic study of 120 cases. Cancer.

[2] Ferner RE, Gutmann DH (2002). International consensus statement on malignant peripheral nerve sheath tumors in neurofibromatosis. Cancer Res.

[3] Weiss S, Goldblum J (2008). Soft tissue tumours.

[4] Kroep JR, Ouali M, Gelderblom H, Le Cesne A, Dekker TJ, Van Glabbeke M (2011). First-line chemotherapy for malignant peripheral nerve sheath tumor (MPNST) versus other histological soft tissue sarcoma subtypes and as a prognostic factor for MPNST: an EORTC soft tissue and bone sarcoma group study. Ann Oncol.

[5] Zehou O, Fabre E, Zelek L, Sbidian E, Ortonne N, Banu E (2013). Chemotherapy for the treatment of malignant peripheral nerve sheath tumors in neurofibromatosis 1: a 10-year institutional review. Orphanet J Rare Dis..

[6] Guo J, Grovola MR, Xie H, Coggins GE, Duggan P, Hasan R (2017). Comprehensive pharmacological profiling of neurofibromatosis cell lines. Am J Cancer Res..

[7] Ambrosini G, Cheema HS, Seelman S, Teed A, Sambol EB, Singer S (2008). Sorafenib inhibits growth and mitogen-activated protein kinase signaling in malignant peripheral nerve sheath cells. Mol Cancer Ther.

[8] Dodd RD, Mito JK, Eward WC, Chitalia R, Sachdeva M, Ma Y (2013). NF1 deletion generates multiple subtypes of soft-tissue sarcoma that respond to MEK inhibition. Mol Cancer Ther.

[9] Fischer-Huchzermeyer S, Dombrowski A, Wilke G, Stahn V, Streubel A, Mautner VF (2017). MEK inhibitors enhance therapeutic response towards ATRA in NF1 associated malignant peripheral nerve sheath tumors (MPNST) in-vitro. PLoS ONE.

[10] Ahsan S, Ge Y, Tainsky MA (2016). Combinatorial therapeutic targeting of BMP2 and MEK-ERK pathways in NF1-associated malignant peripheral nerve sheath tumors. Oncotarget..

[11] Semenova G, Stepanova DS, Dubyk C, Handorf E, Deyev SM, Lazar AJ (2017). Targeting group I p21-activated kinases to control malignant peripheral nerve sheath tumor growth and metastasis. Oncogene.

[12] Sweeney EE, Burga RA, Li C, Zhu Y, Fernandes R (2016). Photothermal therapy improves the efficacy of a MEK inhibitor in neurofibromatosis type 1-associated malignant peripheral nerve sheath tumors. Sci Rep..

[13] Varin J, Poulain L, Hivelin M, Nusbaum P, Hubas A, Laurendeau I (2016). Dual mTORC1/2 inhibition induces anti-proliferative effect in NF1-associated plexiform neurofibroma and malignant peripheral nerve sheath tumor cells. Oncotarget..

[14] Watson AL, Anderson LK, Greeley AD, Keng VW, Rahrmann EP, Halfond AL (2014). Co-targeting the MAPK and PI3 K/AKT/mTOR pathways in two genetically engineered mouse models of schwann cell tumors reduces tumor grade and multiplicity. Oncotarget..

[15] Jessen WJ, Miller SJ, Jousma E, Wu J, Rizvi TA, Brundage ME (2013). MEK inhibition exhibits efficacy in human and mouse neurofibromatosis tumors. J Clin Invest..

[16] Maki RG, D’Adamo DR, Keohan ML, Saulle M, Schuetze SM, Undevia SD (2009). Phase II study of sorafenib in patients with metastatic or recurrent sarcomas. J Clin Oncol.

[17] Fischer-Huchzermeyer S, Dombrowski A, Hagel C, Mautner VF, Schittenhelm J, Harder A (2017). The cellular retinoic acid binding protein 2 promotes survival of malignant peripheral nerve sheath tumor cells. Am J Pathol.

[18] Fischer-Huchzermeyer S. The role of retinoic acid and CRABP2 in NF1-associated peripheral nerve sheath tumours. Thesis, University of Muenster, Germany; 2014.

[19] Aass N, De Mulder PH, Mickisch GH, Mulders P, van Oosterom AT, van Poppel H (2005). Randomized phase II/III trial of interferon Alfa-2a with and without 13-cis-retinoic acid in patients with progressive metastatic renal cell Carcinoma: the European Organisation for Research and Treatment of Cancer Genito-Urinary Tract Cancer Group (EORTC 30951). J Clin Oncol.

[20] Cimino G, Lo-Coco F, Fenu S, Travaglini L, Finolezzi E, Mancini M (2006). Sequential valproic acid/all-trans retinoic acid treatment reprograms differentiation in refractory and high-risk acute myeloid leukemia. Cancer Res.

[21] Huang S, Laoukili J, Epping MT, Koster J, Holzel M, Westerman BA (2009). ZNF423 is critically required for retinoic acid-induced differentiation and is a marker of neuroblastoma outcome. Cancer Cell.

[22] Holzel M, Huang S, Koster J, Ora I, Lakeman A, Caron H (2010). NF1 is a tumor suppressor in neuroblastoma that determines retinoic acid response and disease outcome. Cell.

[23] Frahm S, Mautner VF, Brems H, Legius E, Debiec-Rychter M, Friedrich RE (2004). Genetic and phenotypic characterization of tumor cells derived from malignant peripheral nerve sheath tumors of neurofibromatosis type 1 patients. Neurobiol Dis.

[24] Holtkamp N, Okuducu AF, Mucha J, Afanasieva A, Hartmann C, Atallah I (2006). Mutation and expression of PDGFRA and KIT in malignant peripheral nerve sheath tumors, and its implications for imatinib sensitivity. Carcinogenesis.

[25] Lee PR, Cohen JE, Tendi EA, Farrer R, Dev GH, Becker KG (2004). Transcriptional profiling in an MPNST-derived cell line and normal human Schwann cells. Neuron Glia Biol..

[26] Spyra M, Kluwe L, Hagel C, Nguyen R, Panse J, Kurtz A (2011). Cancer stem cell-like cells derived from malignant peripheral nerve sheath tumors. PLoS ONE.

[27] Franken NA, Rodermond HM, Stap J, Haveman J, van Bree C (2006). Clonogenic assay of cells in vitro. Nat Protoc.

[28] Armstrong JL, Taylor GA, Thomas HD, Boddy AV, Redfern CP, Veal GJ (2007). Molecular targeting of retinoic acid metabolism in neuroblastoma: the role of the CYP26 inhibitor R116010 in vitro and in vivo. Br J Cancer.

[29] Patel AV, Eaves D, Jessen WJ, Rizvi TA, Ecsedy JA, Qian MG (2012). Ras-driven transcriptome analysis identifies aurora kinase A as a potential malignant peripheral nerve sheath tumor therapeutic target. Clin Cancer Res.

[30] Demestre M, Terzi MY, Mautner V, Vajkoczy P, Kurtz A, Pina AL (2013). Effects of pigment epithelium derived factor (PEDF) on malignant peripheral nerve sheath tumours (MPNSTs). J Neurooncol.

[31] Antoszczyk S, Spyra M, Mautner VF, Kurtz A, Stemmer-Rachamimov AO, Martuza RL (2014). Treatment of orthotopic malignant peripheral nerve sheath tumors with oncolytic herpes simplex virus. Neuro Oncol..

[32] Mahller YY, Vaikunth SS, Ripberger MC, Baird WH, Saeki Y, Cancelas JA (2008). Tissue inhibitor of metalloproteinase-3 via oncolytic herpesvirus inhibits tumor growth and vascular progenitors. Cancer Res.

[33] Demestre M, Herzberg J, Holtkamp N, Hagel C, Reuss D, Friedrich RE (2009). Imatinib mesylate (Glivec) inhibits Schwann cell viability and reduces the size of human plexiform neurofibroma in a xenograft model. J Neurooncol.

[34] Li S, Gao Y, Pu K, Ma L, Song X, Liu Y (2011). All-trans retinoic acid enhances bystander effect of suicide-gene therapy against medulloblastomas. Neurosci Lett.

[35] Siddikuzzaman, Berlin GV (2013). Evaluation of immunomodulatory and antitumor activity of all trans retinoic acid (ATRA) in solid tumor bearing mice. Immunopharmacol Immunotoxicol.

