# Supplementary material for: Testing ATRA and MEK inhibitor PD0325901 effectiveness in a nude mouse model for human MPNST xenografts
Source: BMC Res Notes. 2018 Jul 28;11:520. doi: 10.1186/s13104-018-3630-0 (PMC6064132; doi:10.1186/s13104-018-3630-0)
Supplement: Supplementary file 1 — Additional file 1. Mean tumor volumes. Description of data: Mean volumes of transplanted MPNST (mm³) of two treatment groups (mice treated with ATRA and with MEKi) and of placebo group. Tumors were measured from day 7 until day 68 post transplantation. Treatment started at day 42. Average volumes and standard deviation over the whole group per day as well as difference of volumes between day 42 and 68 are given. [file 13104_2018_3630_MOESM1_ESM.pdf]

Group 1 = placebo
Group 3 = ATRA
Group 4 = MEKi

Placebo n=10

	Mouse 5	Mouse 6	Mouse 7	Mouse 8	Mouse 9	Mouse 10	Mouse 11	Mouse 12	Mouse 13	Mouse 14				
**Days po**	**Mean tumor volu**	**Mean tumor volu**	**Mean tumor volu**	**Mean tumor volu**	**Mean tumor volu**	**Mean tumor volu**	**Mean tumor volu**	**Mean tumor volu**	**Mean tumor volu**	**Mean tumor volume (mm³)**		STDEV	AVERAGE VOLUME	
7	107,5361929	141,4093685	154,2396329	105,7135456	93,51735932	161,634942	88,46096594	89,31652634	118,019164	95,26565563		27,43756156	115,5113353	
9	114,9503516	105,7135456	114,7246805	96,87624546	118,7501079	96,87624546	87,11374629	46,72176594	102,0682509	102,1604043		20,6502666	98,5955344	
12	90,16213836	61,60087235	115,1713103	73,62217664	125,4437947	79,26238265	54,28672105	92,38114997	71,27488333	76,48102595		22,43670644	83,96864553	
14	83,9459737	55,49309263	72,45769296	50,66760632	102,6483984	91,13236689	61,28461869	57,36076947	96,61025728	98,63763174		19,91035872	77,02384081	
16	51,8739779	49,85707541	68,96424193	48,7491404	104,9166283	87,02630529	73,62217664	58,36869711	123,8834703	77,95181491		24,95923056	74,52135282	
19	79,19431481	54,28672105	58,90486225	62,83185307	100,6356847	96,87624546	103,6725576	65,97344573	112,7119668	79,19431481		20,86908864	81,42819663	
22	100,6356847	70,79055446	65,68023041	65,44984695	96,87624546	101,3121743	137,1566993	72,90589351	132,7322896	79,39432954		26,359533	92,29339482	
26	93,51893011	64,14085001	58,56085786	80,77820111	103,2604853	83,74229377	65,37025994	32,38563147	94,92845802	65,65300327		21,15273713	74,23389709	
29	61,52285613	98,63763174	84,38946186	68,09402077	77,93244176	88,66831105	88,66831105	45,84212	115,45353	86,13178915		19,55652574	81,53404735	
33	76,34070148	87,02630529	70,45545124	67,95893228	141,3716694	113,0020406	101,3121743	37,75723131	116,2137954	67,95893228		30,21985393	87,93972336	
35	99,75184994	72,06865907	54,47521661	83,9459737	137,1069574	152,7782687	91,59208662	55,43602037	144,3169125	73,97822381		36,28792997	96,54501687	
37	64,71471427	68,09402077	71,6283125	81,65627625	129,2739197	107,4424688	87,28286869	61,64851984	137,626891	82,3620874		26,88254957	89,17300792	
40	66,52322444	88,06931406	81,82906385	81,65627625	99,75184994	98,6543869	108,0645041	52,54418433	153,585658	70,59787011		27,9084725	90,1276332	
42	70,45545124	130,0053872	51,47761362	73,28707342	128,2817	98,01769079	78,81627649	77,42716894	124,6426885	43,58855087		31,36731631	87,59996011	37,85432398
44	72,24825345	103,908177	71,6283125	73,62217664	103,4191358	96,61025728	70,59787011	49,33400024	138,4264263	62,83185307		26,08171661	84,26264624	
47	89,30971956	80,92114357	101,687071	111,7590171	107,756628	125,8982784	87,11374629	82,46680716	143,3508728	62,64649911		23,78915514	99,2909783	
48	79,39432954	106,6738258	67,95893228	86,30792777	87,48707222	77,61042851	76,02654222	61,94173515	164,200576	63,71149901		30,08652785	87,13128685	
51	98,01769079	118,7501079	85,38429953	108,3980365	104,6611121	77,17427073	79,16813487	88,88403375	114,6890758	44,53207586		22,07789827	91,96588378	
54	90,47786842	105,9863406	78,49583404	112,2208312	89,62230802	114,2989947	71,27488333	81,82330426	149,7220227	56,69946421		26,4771668	95,06218515	
56	88,46096594	97,4155522	101,0687009	100,399018	99,71415082	90,31031682	64,14085001	84,46276569	128,3518623	62,64649911		19,06745724	91,69706818	
58	90,07574456	66,34572445	89,31652634	96,60030891	119,4590607	118,4553218	101,3121743	123,6719364	109,3776898	60,34371169		21,69481512	97,4958199	
61	91,43919578	97,4155522	66,48447813	68,09402077	142,3031516	116,3771583	118,4553218	110,85738	109,6855659	71,44924172		25,03535327	99,25610662	
63	65,65300327	63,24392531	70,79055446	96,60030891	173,1923378	107,1571074	89,83070034	107,1927121	124,8594584	57,19897744		35,29684806	95,57190854	
65	78,81627649	81,65627625	75,98413072	72,85876962	88,55940251	83,35744867	89,30971956	97,65902563	141,3716694	90,31031682		19,52047097	89,98830357	
68	80,45828225	101,787602	76,89205099	97,4155522	81,82906385	65,37025994	85,20470515	127,4434184	126,6941485	64,14085001		22,52187506	90,72359333	
Diff68-42	-10,00283101	28,2177852	-25,41443737	-24,12847878	46,45263615	32,64743085	-6,38842866	-50,01624946	-2,05146	-20,55229914	MW Diff	-3,123633222	30,22058012	

ATRA n=9

	Mouse 25	Mouse 26	Mouse 27	Mouse 28	Mouse 29	Mouse 30	Mouse 31	Mouse 32	Mouse 33	Mouse 34			
**Days po**	**Mean tumor volu**	**Mean tumor volu**	**Mean tumor volu**	**Mean tumor volu**	**Mean tumor volu**	**Mean tumor volu**	**Mean tumor volu**	**Mean tumor volu**	**Mean tumor volu**	**Mean tumor volume (mm³)**		STDEV	AVERAGE VOLUME
7		162,2150895	122,7755353	162,0354951	171,5649928	134,9444944	102,6483984	150,1094858	93,3534729	128,8461395		27,35190806	136,4992337
9		139,3689041	139,3689041	167,0212026	197,8386558	114,9503516	110,1426676	114,7246805	95,26565563	148,2517573		31,98006924	136,3258644
12		145,726964	152,7782687	130,8242957	126,9234848	111,0537295	58,90486225	80,77820111	56,57222971	73,53944803		37,35834967	104,1223871
14		96,60030891	109,1054185	72,85876962	90,5721162	83,9459737	88,46096594	73,28707342	25,80504206	76,48102595		23,36192794	79,6796327
16		110,1426676	100,399018	77,86751551	98,63763174	67,95893228	91,13236689	79,19431481	79,39432954	54,28672105		17,45923015	84,33483305
19		80,92114357	117,9563322	49,85707541	74,44265592	82,10028801	86,75979352	64,93933814	60,30234739	68,70663133		19,50937485	76,22062283
22		96,87624546	107,2330292	79,19431481	89,36103224	93,3534729	135,1136168	82,10028801	112,7119668	73,53944803		19,24825374	96,60926825
26		91,95232258	98,42295624	62,64649911	97,4155522	122,6116489	122,222615	80,77820111	66,54312119	89,30971956		21,10159691	92,43362621
29		76,34070148	113,0973355	79,19431481	103,6725576	109,3274243	91,59208662	80,77820111	73,53944803	89,83070034		14,78947826	90,81919664
33		91,95232258	132,9689563	112,2208312	112,7119668	90,31031682	79,19431481	88,06931406	96,87624546	109,1054185		16,59990321	101,4899652
35		82,3620874	105,5575132	120,1009928	123,4771577	96,1327352	73,53944803	82,78515521	46,65265091	64,12618925		25,52040842	88,30376997
37		120,7628216	113,0020406	120,7628216	124,6898124	109,3274243	114,2989947	120,7628216	62,83185307	88,06931406		20,21872084	108,278656
40		192,7178597	148,0773989	130,5200848	110,6102413	103,6725576	97,33701238	111,2123799	91,57061907	96,60030891		32,57234993	120,257607
42		124,788249	94,77766157	111,2123799	101,687071	109,1054185	98,71826595	121,6712655	88,46096594	105,5575132		11,93913586	106,2198656
44		88,06931406	110,6992531	93,51735932	74,81388745	85,38429953	110,85738	107,2330292	63,71149901	81,65627625		16,52750859	90,66025532
47		125,4437947	103,2604853	90,16213836	119,656981	88,66831105	95,12480756	143,675504	70,59787011	88,66831105		22,70167479	102,806467
48		107,4424688	102,1604043	105,5575132	74,85158656	105,7135456	106,1858317	160,5647061	99,75184994	100,6356847		22,42697153	106,9848434
51		110,1426676	114,2989947	137,2519942	86,30792777	98,63763174	89,74273574	94,59754359	42,10153035	86,70795724		25,90372965	95,53210921
54		97,6736864	104,6611121	80,45828225	91,86331078	93,3534729	66,93267868	103,908177	63,24392531	67,65629219		16,35643875	85,52788196
56		79,19431481	116,7373942	67,95893228	87,48707222	91,86331078	76,89205099	93,3534729	43,58855087	68,04689688		20,45054055	80,56911066
58		105,5575132	118,4553218	87,02630529	79,95510383	67,95893228	70,44497927	77,50099637	54,05790839	144,5855187		28,40581395	89,50473101
61		111,5223504	134,9444944	103,908177	97,4155522	114,7246805	104,6998584	73,53944803	77,17427073	119,656981		19,57468408	104,1762014
63		170,2057304	133,9747895	102,0682509	84,82300165	105,5575132	92,07850988	103,908177	60,34371169	103,8908983		30,9340227	106,3167314
65		134,5669797	114,2989947	134,9444944	62,29568793	112,8224462	94,72844328	112,8208754	68,09402077	85,66442488		26,40229277	102,2484853
68		63,71149901	128,8136764	83,9459737	99,75184994	79,39432954	84,53397512	105,5575132	113,7926747	104,6611121		19,90231098	96,01806708
Diff68-42		61,07674999	-34,03601483	27,2664062	1,93522106	29,71108896	14,18429083	16,1137523	-25,33170876	0,8964011	MW Diff	10,20179854	28,90810088

MEKi n=10

	Mouse 35	Mouse 36	Mouse 37	Mouse 38	Mouse 39	Mouse 40	Mouse 41	Mouse 42	Mouse 43	Mouse 44			MEKi
**Days po**	**Mean tumor volu**	**Mean tumor volu**	**Mean tumor volu**	**Mean tumor volu**	**Mean tumor volu**	**Mean tumor volu**	**Mean tumor volu**	**Mean tumor volu**	**Mean tumor volu**	**Mean tumor volume (mm³)**		STDEV	AVERAGE VOLUME
7	166,76621	110,6102413	164,9336143	133,9747895	103,9217906	124,788249	124,6898124	101,787602	172,9959883	72,06865907		32,73613358	127,6536956
9	136,3252244	119,9810886	114,4005729	99,54921721	87,28286869	99,90264638	126,767976	117,2861257	118,4553218	73,28707342		19,11445021	109,3238115
12	74,44265592	99,54921721	76,02654222	94,59754359	57,82939037	69,45590118	166,76621	97,66007283	69,37474337	60,87987684		31,83299617	86,65821535
14	98,63763174	82,0191302	97,22129705	81,02376893	79,19431481	98,01769079	79,26238265	81,8515786	87,67399698	82,3620874		8,099004424	86,72638792
16	124,5730499	115,45353	81,05309046	55,49309263	69,7480693	69,37474337	64,40683819	118,7757643	60,18767926	59,88713356		27,02107345	81,8952991
19	132,1212498	92,28742579	81,43008158	38,77772532	65,1712924	69,12708115	78,49583404	100,6356847	103,6725576	68,96424193		25,65403094	83,06831743
22	148,2517573	106,0873951	100,4492835	47,71293843	91,66696124	104,1081917	80,19648287	98,01769079	124,6898124	63,35545185		28,69086713	96,45359652
26	174,463112	102,62536	117,8097245	84,54654149	102,62536	101,761422	120,5334853	84,46276569	66,93267868	49,74188368		33,98850533	100,5502333
29	203,2217748	96,84482953	147,7805184	84,82300165	88,48819308	100,6356847	150,1094858	92,91260273	42,41150082	73,97822381		46,19554608	108,1205815
33	173,7175074	130,2650922	265,9044022	105,8591061	79,16813487	82,52126143	129,33361	120,7549676	89,30971956	104,6611121		55,92814655	128,1494913
35	171,897478	139,6987713	186,6341656	84,54654149	96,1327352	84,82300165	123,1629984	103,0835089	77,50099637	83,12654161		39,16281846	115,0606739
37	194,3860454	130,2650922	117,9479546	131,1421201	110,1426676	79,19431481	110,6992531	96,23693136	63,71149901	86,54668882		36,25886081	112,0272567
40	209,3075633	93,75350236	124,2374231	98,71826595	90,31031682	108,1200056	102,62536	92,23558951	68,96424193	92,91260273		38,23554459	108,1184871
42	229,0598036	97,22129705	118,7757643	59,46406575	97,33701238	66,73214035	115,034651	91,75126065	49,46123474	66,93267868		51,32533585	99,17699085
44	141,4093685	94,61848754	161,4605836	68,09402077	84,53397512	113,7926747	63,79265683	88,48819308	66,54312119	60,75421313		34,56434653	94,34872945
47	194,9714289	82,10028801	114,8644814	56,28686838	108,3980365	84,53397512	68,04689688	76,00140948	74,44265592	88,95105439		39,28450633	94,8597095
48	117,2468558	76,54438141	87,93108398	102,2070046	87,28286869	91,59208662	84,7570282	74,78561312	84,53397512	60,75421313		15,36975722	86,76351107
51		93,75350236	93,75350236	90,07574456	72,73572391	84,54654149	49,33400024	66,53474361	63,71149901	64,93933814		15,74739813	75,4871773
54		59,54103477	84,38946186	112,1784197	118,7757643	56,63244357	58,60379296	133,7857704	90,5721162	104,1081917		28,47507328	90,95411061
56		199,2492309	117,9479546	86,96609144	100,059726	73,97822381	60,18767926	99,3413485	113,7926747	108,3980365		39,47599077	106,6578851
58	235,8854372	130,5425995	146,5364477	151,4970225	150,3178781	110,4704405	102,9389957	126,744414	83,74229377	86,70795724		44,01294908	132,5383486
61	212,0575041	108,6545999	151,4750314	107,1408759	111,7590171	111,7590171	138,4311387	133,7941479	97,49409202	114,8644814		33,65451998	128,7429906
63	175,7862461	73,00275929	130,3855199	133,7941479	110,6102413	56,28686838	121,4152257	120,9078585	86,70795724	133,489937		34,52495264	114,2386761
65	154,9517273	133,9789783	121,0518481	178,6372415	161,4296913	79,16813487	100,4304339	111,2877782	98,01769079	77,20778105		34,94006925	121,6161305
68	173,7693437	140,8485942	106,0873951	114,0021142	121,4152257	92,91260273	77,93244176	123,2237358	62,59937522	70,44497927		34,08858955	108,3235808
Diff68-42	55,2904599	-43,62729715	12,6883692	-54,53804845	-24,07821332	-26,18046238	37,10220924	-31,47247515	-13,13814048	-3,51230059	MW Diff	-12,89113274	28,34269267
